# Improvement of osteogenesis by a uniform PCL coating on a magnesium screw for biodegradable applications

**DOI:** 10.1038/s41598-018-31359-9

**Published:** 2018-09-05

**Authors:** Yu-Kyoung Kim, Kwang-Bok Lee, Seo-Young Kim, Yong-Seok Jang, Jin Hyeok Kim, Min-Ho Lee

**Affiliations:** 1 Department of Dental Biomaterials and Institute of Biodegradable Materials, Institute of Oral Bioscience and School of Dentistry (plus BK21 program), Chonbuk National University, Jeon Ju, 561-756 South Korea; 2 Department of Orthopedic Surgery, Research Institute of Clinical Medicine of Chonbuk National University-Biomedical Research Institute of Chonbuk National University Hospital, Chonbuk National University Medical School, Jeon Ju, 561-756 South Korea

## Abstract

A polymer coating as polycaprolactone (PCL) is applied to improve the initial corrosion resistance of biodegradable magnesium. In addition, plasma electrolytic oxidation (PEO) is performed to increase adhesion between the polymer and the metal. However, when a complex-shaped material such as a screw is implanted in a bone, the surface coatings are locally damaged, and the protective role of the coating is not sufficiently maintained. In this study, the optimal conditions for producing a polymer coating on a screw were determined by varying the concentration of the PCL and the coating cycles, and were examined *in vitro* and *in vivo*. Among various the PCL coating conditions of 2∼6 cycles with 5∼7 wt.% concentrations, the 6 wt.% + 4 cycles group was applied uniformly to the screw thread. In the case of the non-uniform PCL layers, oxides and gases were present between the Mg and the PCL layer because internal magnesium corrosion and the layer peel off. The 6 wt.% + 4 cycles group had a high corrosion resistance due to the low wear on the thread. Denser and thicker bone formed around the PCL-coated screw in rat femur. This difference was due to the high corrosion resistance, which provided sufficient time for bone healing and promoting new bone growth.

## Introduction

Biodegradable polymer coatings on magnesium (Mg) and its alloys improve the corrosion resistance of implants in the body over the long term and improves the biocompatibility of metallic Mg in biodegradable applications^1–3^. The important requirements for the coating to achieve the desired performance are uniformity, lack of porosity, biocompatibility and self-healing ability^4,5^. Natural biodegradable polymers such as collagen, polysaccharides like chitosan, and synthetic biodegradable polymers such as polycaprolactone (PCL) and poly-L-lactide (PLLA) provide good protection and have exhibited biocompatibility on Mg^6–8^. These biopolymers can be blended, their surfaces can be modified, and the materials can be loaded with growth factors to adjust their bioactivity, mechanical properties, drug release and degradation under physiological conditions. Among the biopolymers, PCL is produced from the ring-opening polymerization of the cyclic monomer ɛ-caprolactone^9^, this polymer has a slower degradation rate than PLLA and has also been applied as a surface coating on magnesium alloys. Dip coating, air spraying, electrospraying and spin coating, etc. are used as simple methods for coating a polymer on a metallic material^10–13^.

However, tailoring the degradation period of the biodegradable polymer coating requires careful selection of the polymer type and layer thickness. Furthermore, there are limits to the formation of uniform coatings on medical devices with complex and elaborate three-dimensional structures due to the low adhesion stability. It is difficult to achieve long-term corrosion protection using a thin biodegradable polymer coating, while an increase in the coating thickness could lead to cohesive failure. To solve this problem, the biodegradable polymer consisting of monomers need an initiator such as hydroxyl groups for ring-opening polymerization^14,15^. The methods of hydroxyl groups formation on metal are typically nitrogen plasma treatment, binding of a chemical substance with a hydroxyl group in the molecule^16,17^. One of the other methods is physical modification by deposition of an oxide film under the PCL layer on Mg and its alloys.

However, non-uniform oxide films suffer from the presence of pores and cracks in the coating that seriously impede their corrosion protection abilities^18^. Plasma electrolytic oxidation (PEO) with a pulsed source is a type of electrochemical surface treatment method that forms a homogeneous oxide layer in the plasma state that is generated by applying an extremely high voltage in a suitable electrolyte^19–21^. In this study, the PEO treatment was used to improve the bonding strength between Mg and the PCL layer by increasing the surface area dramatically. A previous study showed strong adhesion and a high corrosion resistance for the PCL layer when the surface was modified by anodic oxidation^6,18,22^. In addition, the film thickness and corrosion resistance increased when the PCL concentration in the coating solution was high, but this case was examined for only flat specimen^18,23^. Medical devices have complicated and irregular shapes. Although there have been studies on forming polymer coatings on unusual shapes such as biodegradable scaffolds for improving the biocompatibility in the case of metallic magnesium^24^, a non-uniform coating results in local corrosion. Therefore, the formation of a uniform PCL coating on a commercial device with a complex shape would require different conditions, such as the concentration of solute, dipping time and number of deposition cycles. In this study, the PCL coating produced under the optimal conditions on a Mg plate was applied to the anodized magnesium screw, as described in a previous study, and then we aimed to find the optimal number of cycles and concentration for producing uniform PCL layers on complex materials such as orthopaedic absorbent fixtures by examining the immersion of an artificial bone model plate in simulated body fluid and performing *in vivo* studies in a rat tibia.

## Results

**Figure 1:**
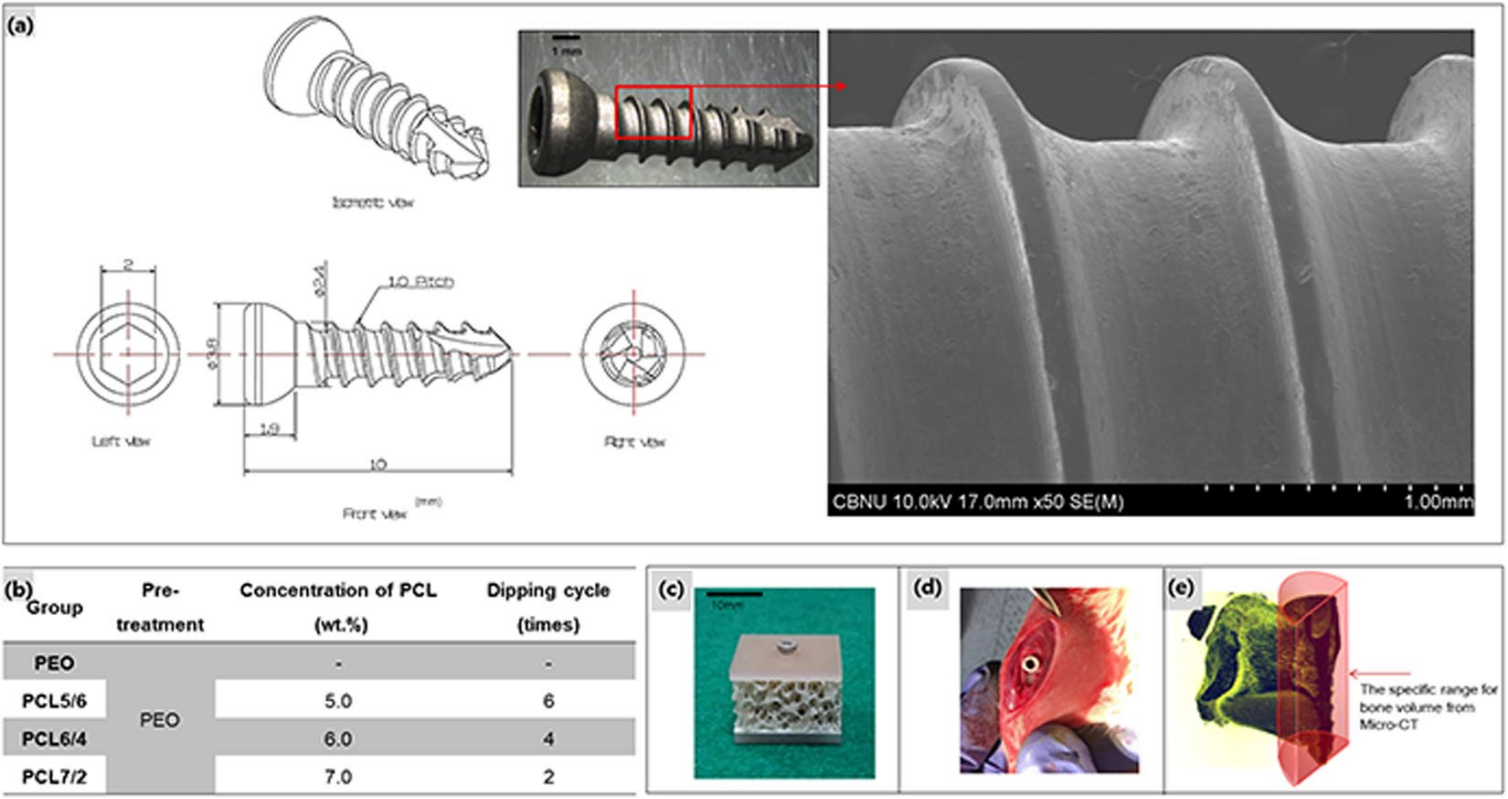
(**a**) Design of the screw and surface morphology; (**b**) Conditions of coating the PEO Mg screw by PCL, (**c**) artificial bone plate model for the immersion test, (**d**) *in vivo* model in a rat tibia, and (**e**) bone volume measurement range.

**Figure 2:**
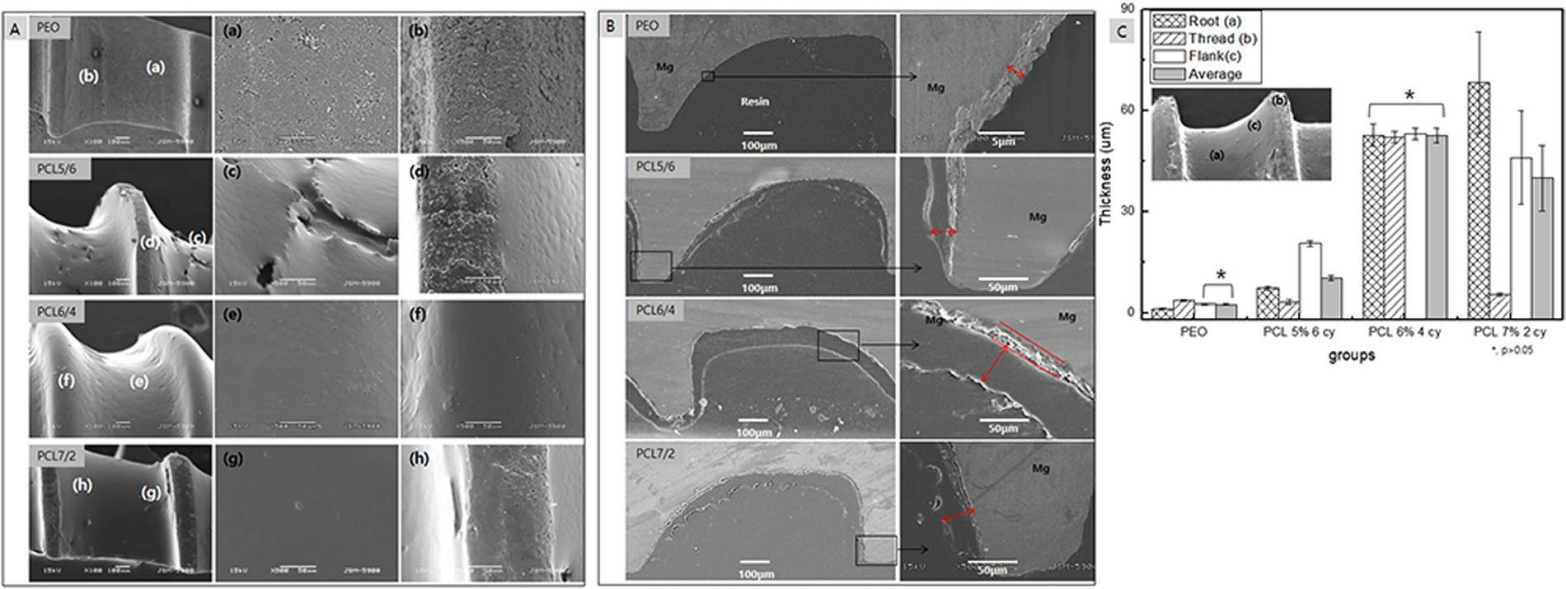
(**A**) The surface morphologies of PCL coatings prepared under various treatment conditions, (**B**). The cross-sectional images of the PCL and PEO coatings and (**C**). Thickness of the films at various points.

An uneven polymer coating on a screw is stripped from the threaded portion, when the screw is inserted into bone. To solve this problem, the surface of a magnesium screw was treated with PEO to increase the surface area and hydrophilicity, thereby allowing a uniform and stable PCL layer to be formed. A bone screw was manufactured as shown in Fig. 1(a), and PEO coating was performed using a pulsed power source. As a result of observing the manufactured screw with FE-SEM (Fig. 1(a)), threads and roots of screw were processed in accordance with the design, and there were not shown large machining defects, chips or foreign matter on the surface. A typical porous oxide film having pores less than 3 μm in diameter was obtained, as shown in Fig. 2A(a,b). Then, the polymer layer was formed on the PEO layer by varying the concentration of PCL and the number of coating cycles. In our previous study, it was found that the PCL coating formed from the 7 wt.%-2 cycles conditions on the PEO layer was thicker and had a higher corrosion resistance than the PCL coating produced from the 4 wt.%-2 cycles conditions. Therefore, when these optimum conditions were applied to the screw-shaped specimen, the screw root portion was uniformly covered, as shown in Fig. 2A(g), whereas the thread portion was not uniformly coated with PCL, and the oxide layer remained partially exposed, as shown in Fig. 2A(h). Therefore, we improved the uniformity of the coating by lowering the PCL concentration and increasing the number of cycles. As shown in Fig. 2A(f,e), the PCL6/4 layer uniformly covered the surface of the thread and the root of the screw. On the other hand, the screw surface of the PCL5/6 sample was covered by a thick polymer layer that was not uniform, where a bark-like coating layer was observed, and the threaded portion was rather thinly coated, leaving part of the PEO coating exposed, as shown in Fig. 2A(c).

Figure 2B,C showed the cross-sectional images and thicknesses of the various coated screws. A uniform oxide film with a thickness of approximately 2 μm was formed on the entire screw through the PEO treatment. The thickness of the PCL layer on the PCL 5/6 screw thread was thin at approximately 3.61 ± 0.18 μm, but the film on part of the flank (c) was thicker at approximately 20.52 ± 0.85 μm (Fig. 2C). In addition, the film thickness on the PCL 7/2 group showed the greatest variation across positions, where the film on the root portion was thick at 68.0 ± 14.8 μm and that on the thread portion was approximately 5.4 μm thickness. The PCL coating on the PCL 6/4 group exhibited an average thickness of 52.6 μm at all locations and was the uniform most coating to statistical significance, as shown in Fig. 2C.

**Figure 3:**
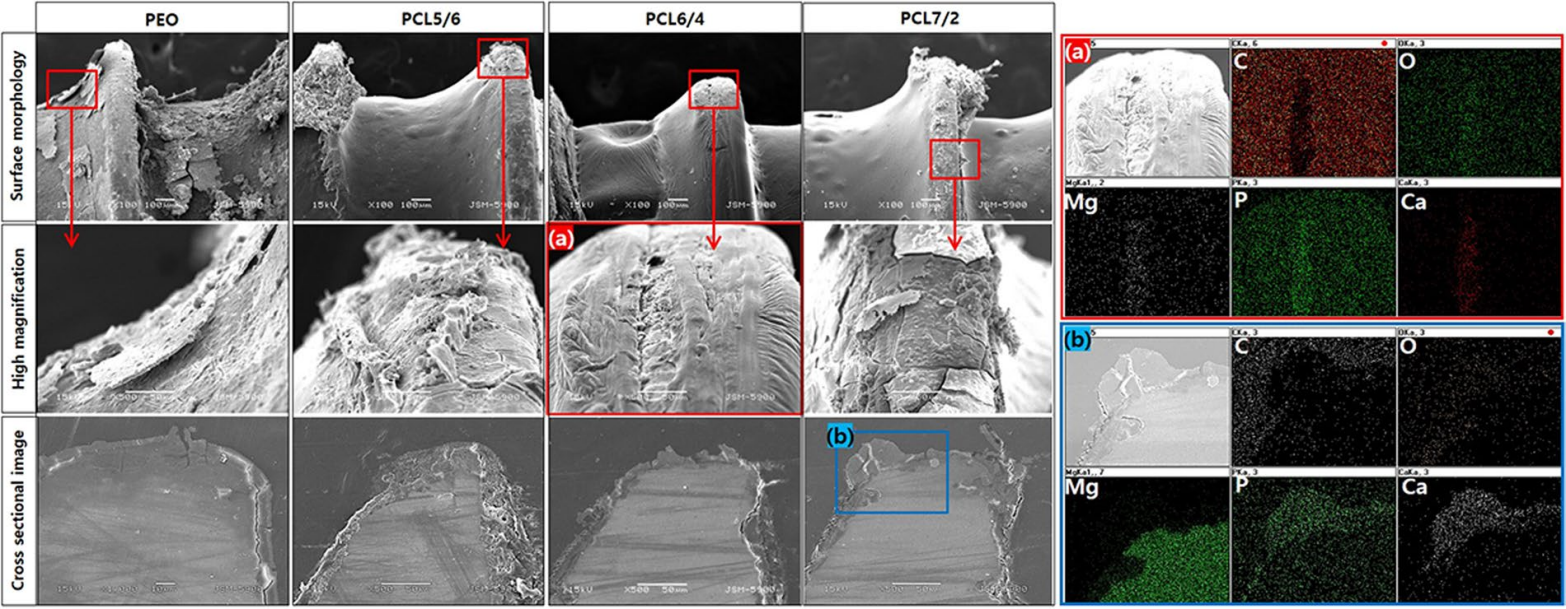
Surface morphologies and cross-sectional images after immersion for 1 month and elemental composition determined by mapping of regions (**a**,**b**).

To simulate the damage process of the coated screw during implantation in the actual bone environment, an open-cell rigid foam and two short fibre-filled epoxy sheet similar in structure and mechanical properties to cancellous bone were manufactured, and the coated screws were implanted in the artificial bone sheet for immersion tests in SBF (Fig. 1c). Figure 3 shows the surface morphologies of the various coated screws after being implanted in the artificial bone model plate and immersed in Hank’s solution for 1 month. The PEO-treated screw was completely corroded, and the oxide layer was broken and had peeled off. It was also confirmed that cracks formed between the magnesium substrate and the oxide film in the cross-sectional image, and the irregular corrosion product partially penetrated into the substrate. In the PCL5/6 group, the thin and unstably-coated PCL layer peeled off at the thread portion, leading to the corrosion of the exposed PEO layer and inner magnesium substrate. The PCL6/4 group was uniformly formed, but the PEO layer was partially exposed, as the thread portion was damaged during screw implantation (Fig. 3(a)). From EDX mapping, Mg, O, P and Ca were detected only in the exposed parts, and C was the main element in the PCL polymer. Though the root portion of the PCL7/2 group was not damaged by the thick film and corrosion was not observed, the thread portion was easily peeled off by the insufficient adhesion of the PCL coating, and local corrosion proceeded similarly to that observed in the PCL5/6 group. Magnesium oxide was formed by localized dissolution of the exposed substrate, and the local increase in pH caused by the formation of OH^−^ through local corrosion resulted in reaction with P and Ca in the simulated fluid to induce calcium phosphate binding (Fig. 3(b)).

**Figure 4:**
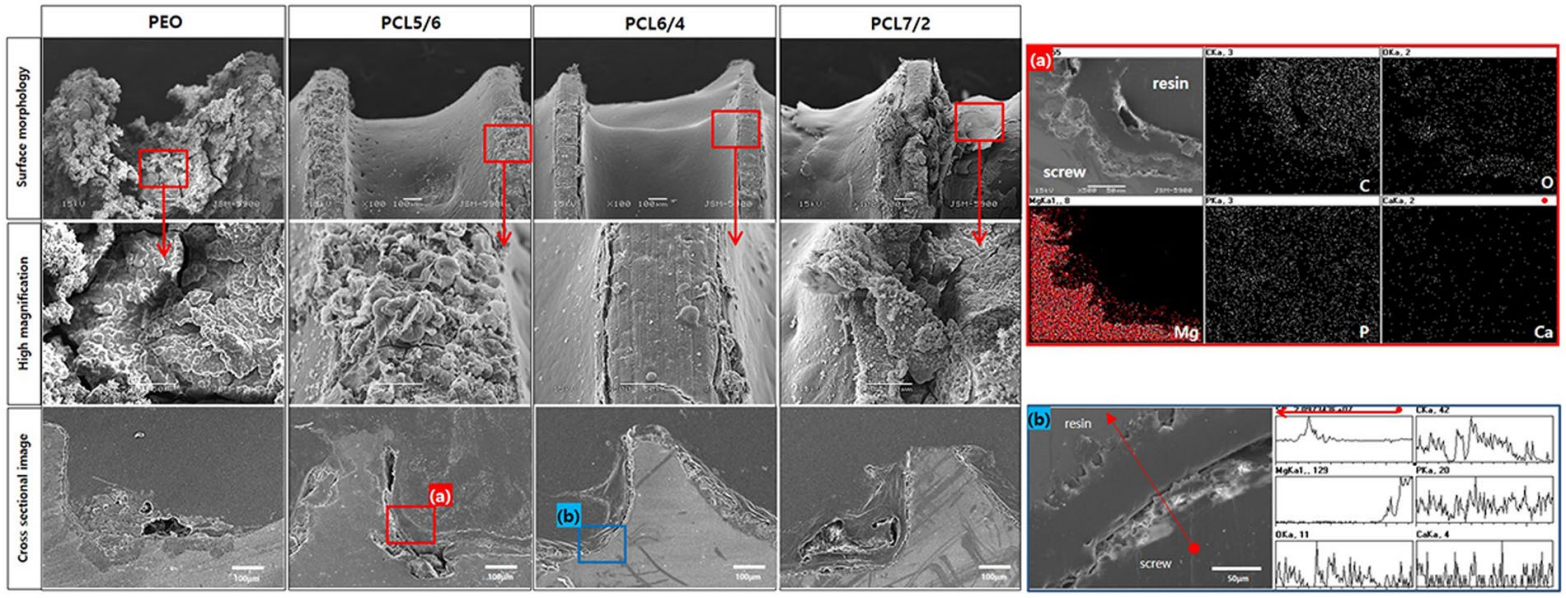
Surface morphologies and cross-sectional images after immersion in SBF for 3 months and elemental composition determined by mapping of region (**a**) and line scanning of region (**b**).

Figure 4 shows the surface morphologies of various coated Mg screws implanted in a hole drilled in the artificial bone model and left for 3 months. In the PEO group, the whole area of the screw corroded, and corrosion products were observed on the surface. Local corrosion occurred in the screw root, and porous corrosion products formed over it based on the cross-sectional image. In the PCL5/6 and PCL7/2 groups, substantial dissolution occurred in the threads of the PCL layer, and the root portion also underwent local corrosion inside the PCL layer, as in the one-month results. Through EDX mapping (Fig. 4(a)) of the screw root of the PCL5/6 group, oxygen was detected between the magnesium substrate and the PCL layer because magnesium corrosion occurred under the existing PCL layer. On the other hand, the PCL6/4 group was damaged during implantation, but local corrosion did not occur, and the coating layer of the threaded portion remained stable. The cross-sectional line scanning result (Fig. 4(b)) also revealed that the PEO layer was maintained under the PCL.

**Figure 5:**
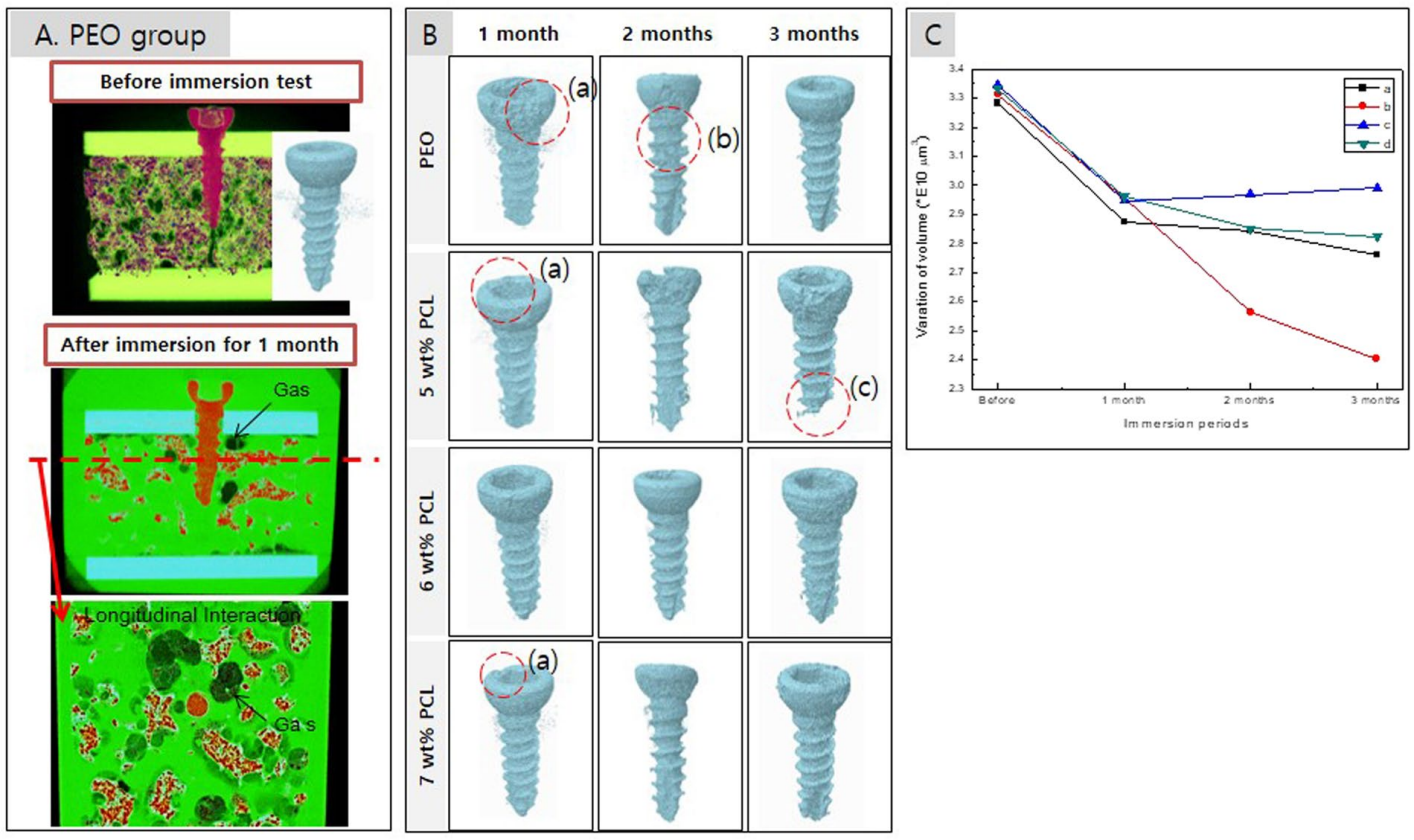
(**A**) Simulation of the screw in the artificial bone block and gas formation of the PEO group; (**B**) 3D images obtained by micro-CT after the immersion test (a) defect of head portion, (b) defect of neck portion and (c) defect of bottom portion; and (**C**) volume variations in the screw after the immersion test (a) PEO, (b) 5 wt% PCL, (c) 6 wt% PCL and (d) 7 wt% PCL.

After immersion for 1 month, micro-CT images of the artificial bones with implanted screws showed that hydrogen gas formed around the screw through the corrosion reaction (Fig. 5A). The gas pocket formed inside the artificial plate was like the formation pattern in the actual cortical bone. In this study, we tried to measure the amount of gas formed using micro CT, but it was not impossible to measure accurately because of the loss of formed gas due to the rapid flow of SBF unlike the *in vivo* situation. Figure 5B shows a CT image of the screw after implantation for 1 to 3 months, where rapid erosion was observed in the screw heads (Fig. 5B(a)) exposed to the SBF. After immersion for 2 months, the PEO group showed the highest corrosion rate at the screw neck, as shown in Fig. 5(b), and the PCL5/6 group showed the fastest corrosion in the head and thread; in particular, the screw tip dissolved after 3 months, as shown in 5B(c). As shown in Fig. 5C, the volumes of all groups decreased at a similar rate after one month. While the volume of the PCL5/6 group decreased significantly, leaving only 70% of the initial volume screw due to the dissolution of the screw tip. The volume of the PCL6/4 group did not decrease even though the screw head corroded but rather showed a slight increase in volume with the formation of the corrosion products.

**Figure 6:**
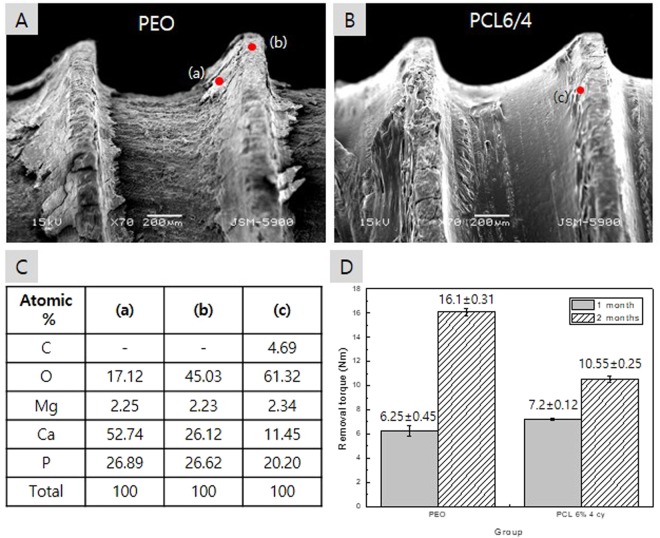
*In vivo* removal torque after 2 months: (**A**) surface morphology of the PEO-coated screw, (**B**) surface morphology of the PEO + PCL coated screw, (**C**) elemental composition of the specified points in figures from (**A**,**B**) and (**D**) mean of the removal torque after 1 and 2 months.

From the above physical and *in vitr*o evaluations, it was confirmed that the coating conditions of 6 wt.% PCL and 4 cycles exhibited the most stable PCL attachment, the least membrane damage and the highest corrosion resistance during implantation. Therefore, the screws of the PCL6/4 group were implanted in rat tibias, and the new bone formation and screw removal torque were evaluated. Figure 6 shows the removal torque for a rat tibia and the surface of the screw after removal. The PEO screw was removed from the bone, and the oxide film was completely removed due to the corrosion of the screw root. The high Ca and P contents detected in the flank sections (Fig. 6(a)) were due to the bone particles from the surrounding bone tissue, and in the thread part (Fig. 6(b)), Ca and P were considered to be part of the corrosion products formed from MgO and SBF. For the PCL-coated screw shown in Fig. 6B, the PCL coating on the root and flank portions was maintained without damage, and the PCL layer on the threads was worn. In the EDX results shown in Fig. 6C(c) for the damaged thread part of the PCL film, carbon from the polymer was observed, and higher oxygen and lower Ca contents than those of the PEO group were detected because of the formation of fewer corrosion products. Otherwise, the removal torque of the PEO group was lower than that of the PCL group after 1 month of implantation but significantly higher than that of the PCL group after 2 months.

**Figure 7:**
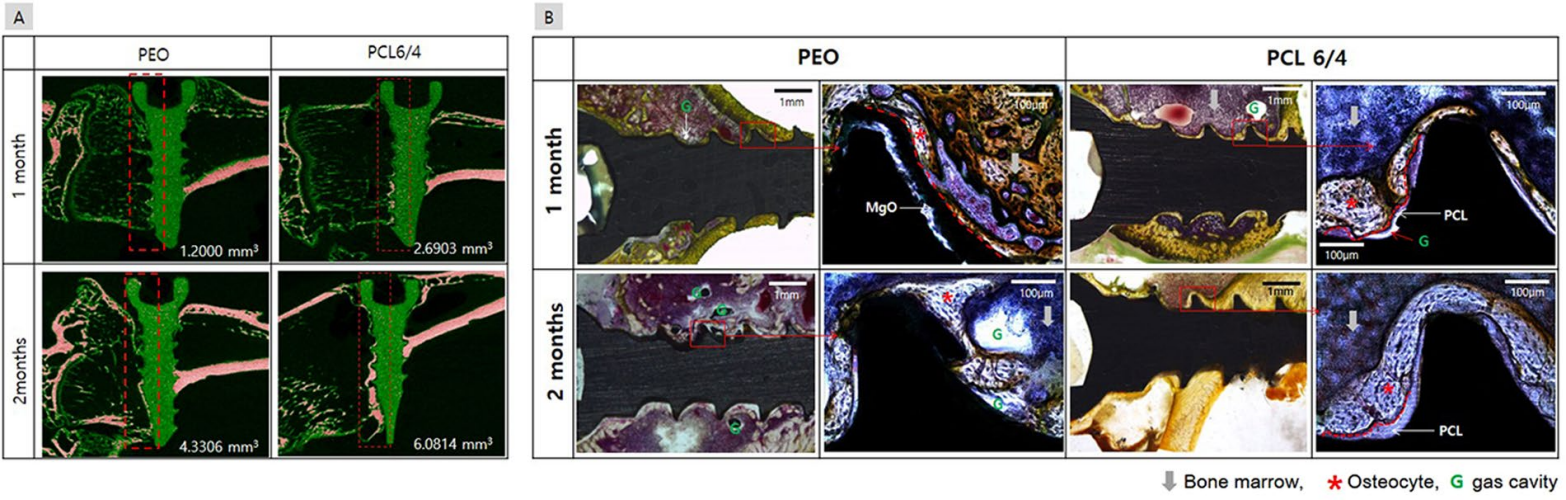
(**A**) Images of the horizontal sections of the tibias and the corresponding bone volumes, and (**B**). Histological analysis of the PEO and PCL coated screws in the tibia after 1 and 2 months.

Figure 7A shows the results of micro-CT evaluations performed to observe new bone formation and magnesium degradation after implantation in a rat tibia. Because the formation of new bone occurred more rapidly in the cancellous bone than in the cortical bone at the implanted position, only the half of the screw inserted into the cancellous bone was scanned and quantified, as shown in Fig. 1(e). The mature bone of the tibia was marked with red, and its volume measured. After 1 month, the volume of bone in the PEO group was 1.25 mm^3^, whereas the volume of bone tissue around the PCL-coated screw was higher with a value of 2.69 mm^3^. After 2 months, the peripheral bone tissue of the PCL-coated screw showed the largest value of 6.081 mm^3^. From the histologic analysis shown in Fig. 7B, some gas cavities were observed in both the PEO and PCL groups after 1 month. In particular, a thick and stable osteocyte was formed around the PCL-coated screw. Gas formation increased in the PEO group as part of the corrosion process of magnesium after 2 months. In addition, erosion of the thread was observed, the inner magnesium substrate dissolved locally to form irregular oxides, and osteoblasts partially formed on them. On the other hand, gas pockets were not observed, and dense new bone cells were formed around the PCL-coated screw. At the time of implantation, the PCL layer was worn, but there was no damage to the inner PEO layer, so new mature bone formation was also observed at the screw head portion.

## Discussion

There are many studies on the corrosion properties and biocompatibilities of ceramic oxides formed on the surfaces of medical magnesium devices using the plasma electrolytic oxidation method^21,25,26^. In addition, it is possible to precisely control the initial corrosion resistance of the absorbent magnesium metal by further coating it with an absorbing polymer such as PCL to seal the pores of the PEO layer^18^. In our previous results^27^, *in vivo* and clinical observation of the non-treated Mg screw for gas formation and absorption were not shown adverse effects such as the inhibition of peripheral tissues growth and toxicity in the liver and kidney, so it is possible to apply the Mg screw itself for medical use without any additional treatment. In this study, we tried to evaluate the change of the corrosion mechanism due to the polymer coating on the metal and the acceleration of the corrosion of magnesium accordingly.

**Figure 8:**
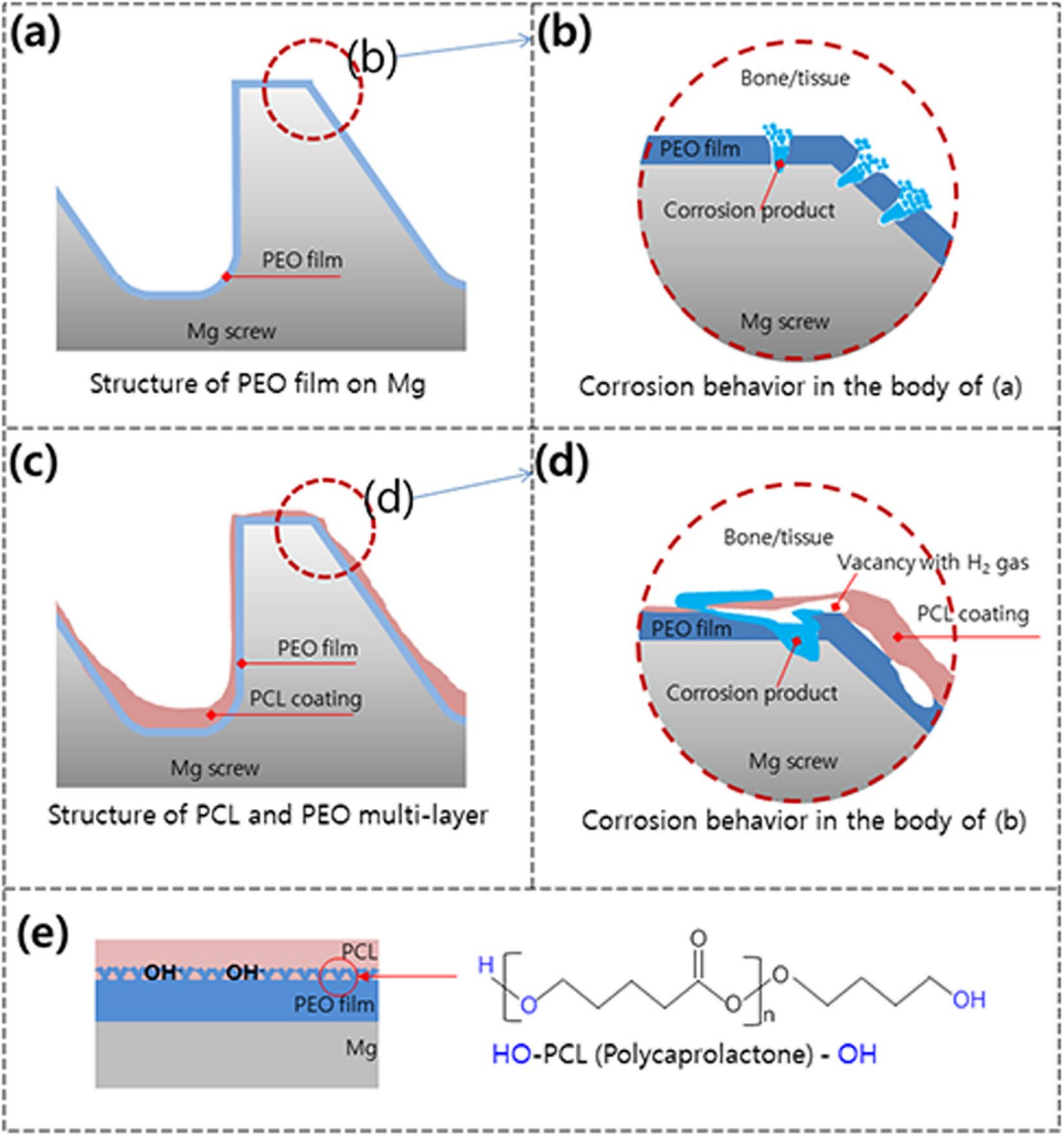
Schematic diagrams of the biocorrosion behavior of the PCL and PEO multilayers formed on the Mg screw: (**a**) PEO film, (**b**) corrosion behavior after implantation of (**a**,**c**) unstable PCL coating, (**d**) corrosion behavior after implantation of (**c**,**e**) the structure between the film and the polymer.

The corrosion mechanism in the body of the PEO and PEO + PCL coatings on magnesium surfaces is illustrated in Fig. 8. The magnesium oxide formed by PEO (Fig. 8(a)) has a ceramic structure with high strength but brittle, and the PCL coating is easily damaged and peeled off when it implanted to the bone, even by the small vacancy caused by gas pocket formation in the polymer layer (Fig. 8(c)), resulting in detachment of the coating from the alloy surface^15^. For this reason, when a complex-shaped material such as a screw is implanted in a bone, the MgO layer and the polymer coating are locally damaged (Fig. 8(d)), and the protective role of the coating is not sufficiently maintained as materials with an ideal flat surface. The polymer coating method on magnesium used in this study induces physical bonding by repetition of dipping coating. In terms of chemistry, the chemical bonding structure is the combination of OH- and PCL structure of anodic oxide film formed on magnesium. Mg(OH)_2_ formed under the PCL polymer coating has a bonding structure with a polymer layer as follow; (Fig. 8(e))

$$(-\mathrm{COO}\,R-)\,\mathop{\longrightarrow }\limits^{{({\rm{Mg}}({\rm{OH}}))}_{2}}\,{\rm{RCOOH}}\,{\rm{or}}\,{\rm{ROH}}$$

It decomposes to acid with unstable Mg (OH)_2_ catalyst (especially amorphous regions).

In this study, the optimal conditions for introducing PCL coatings on screw-shaped specimens were examined by varying the PCL concentration and the number of dipping cycles performed on the PEO layer, and multiple layers were applied to evaluate the bioabsorption rate of the magnesium screw and the *in vitr*o and *in vivo* bone formation. Previous study of Li *et al*., 4 w.t% PCL was applied in 2 cycles to a PEO film to form a thin film with a thickness of ∼600 nm, and the 7 wt.%-2 cycle PCL conditions produced a uniform coating approximately 9 μm thick that had a higher corrosion resistance^18^. Plate-type specimens with smooth surfaces or scaffolds with a low risk of abrasion in the body can be coated with a high PCL concentration to simply for formed a thick PCL coating and increase the corrosion resistance^28^. And it theoretically prevented oxidation of the metal and greatly slowed the absorption. However, when the magnesium is exposed due to the local damage of the polymer, the pitting corrosion between the polymer layer and the magnesium grows, the rapid rise of the pH due to the magnesium corrosion inside, and the peeling of the polymer may accelerate the local decomposition of the magnesium. To apply a uniform film to a complicated medical device such as a screw, various factors of the coating conditions should be considered. In general, the viscosity and surface tension of a solution are proportional to the concentration of PCL in the solvent. Therefore, if the viscosity is too low, it is difficult to form a film with a thickness over a certain limit, and, a coating of non-uniform thickness will form on the curved surface of the screw when the viscosity is too high. The viscosity of the coating solution increases with increasing degree of entanglement, but the surface tension can be controlled by varying the contents of various solvents, such as water, formic acid and dichloromethane^29^. Therefore, in this study, the PCL concentration in dichloromethane was varied in the range of 4∼7 wt.% and applied in 2 to 6 cycles, and the optimum conditions for coating a PEO-treated Mg screw were established. The microscopic hole defects commonly seen in all PCL coating groups are due to the phase separation process, in which solvent evaporation from the polymer solution acts as the force for phase separation, mainly because the polymer solution becomes thermodynamically unstable during evaporation of the solvent^30^.

In the PCL 7/2 group, the PCL layer had a thickness of 68 μm or more in the screw root due to the high viscosity and surface tension of the solution, and the PEO layer on the thread portion was exposed by the non-uniform coating layer. On the other hand, although 6 cycles were applied with the 5 wt.% PCL solution, it was difficult to form a coating having a sufficient thickness because of the low solution viscosity. If the thickness of the biodegradable polymer coating is thin, it is difficult to completely seal the pores of the PEO layer. In addition, the generation of defects and local corrosion easily occur, which makes it difficult to achieve corrosion prevention for a long time. On the other hand, if the thickness of the coating is increased, it is difficult to form a coating of uniform thickness, and the coating layer tends to fall off^31^. The PCL 6/4 coating was uniformly formed on the all surfaces of the screw, including the root and thread. As shown in Fig. 3(a), implantation in the artificial bone plate did not damage the inner PEO layer, although only some of the PCL coating in the thread area was lost.

Initial local corrosion occurred only in the exposed areas of the magnesium substrate, but corrosion under the PEO layer was accelerated in the damaged PCL groups, especially the PCL 7/2 and PCL 5/6 screws, after 3 months of SBF immersion. This mechanism is shown in Fig. 8(c,d); SBF permeated the gaps of the damaged PCL layer and the porous PEO film, causing the magnesium metal to corrode and the surrounding pH to rapidly increase, as follows. The dissolved divalent Mg^2+^ ions reacted with hydroxyl groups (OH^−^) and precipitated as Mg hydroxide (Mg(OH)_2_)^32^. In particular, the erosion rate of biodegradable polymers is faster in alkaline solution because the increase in the hydrolytic rate of alkaline hydrolysis may be the result of random hydrolytic scission of the polymer chain^33^. However, since the degradation rate of the polymer was slower than the corrosion rate of magnesium, the corrosion process penetrated the PCL layer, as shown in Fig. 4(a). In addition, hydrogen gas was generated and collected by the corrosion products of magnesium that accumulated below the PCL coating, causing the PCL coating layer to swell and eventually fall off. That is, although the metal surface was initially well protected by the uniform PCL coating, the thin and non-uniform PCL coating increased the rate of absorption of the magnesium screw by causing the polymer layer to peel following filiform corrosion under the PCL coating layer over time in SBF^31^.

For the screws implanted in an artificial bone block, the head portion that was directly exposed to the SBF dissolved faster than the screw body of the portion embedded in a limited diffusion environment. In the Leonie W study^34^, the degradation behaviour of a Mg screw determined by *in vitro* and animal model studies showed the same pattern, and considerable damage was seen in the head part that was turned by the driver, as shown in Fig. 5B(c). The starting location of the stress and the highest concentration point of the screw were the first threads of the lead next to the screw head region when the screw was implanted in the bone. Regarding the behaviour of stress distribution, it was seen that the higher the applied load was the greater the formation along the screw body. And consequently, the higher the stress in the most important area near the screw head, as shown in Fig. 5B(b)^35^. This distribution also occurred in a prosthetic screw after preload^36^. Even in the axial and oblique load modes, the highest stress was concentrated in the neck and the last thread of the screw^37^.

In previous *in vitro* simulations of this study, it was suggested that a uniform polymer coating on an entire screw, including the threads, could effectively suppress the initial corrosion of the magnesium screw. The conditions necessary for SaOS-2 osteosarcoma cells to attach to the surface and then spread and proliferate to confluence on PCL-coated Mg has also been reported^38^. On the other hand, as shown in Fig. 6D, the removal torque in the rat femur showed higher bone attachment strength for the PEO group than the PCL groups. This difference is due to the increase in the volume of the Mg(OH)_2_/Mg (the Pilling-Bedworth ratio for Mg(OH)_2_ is 1.77)^39^ and the increase in surface roughness and area due to the presence of magnesium oxide and apatite. Therefore, the PCL coating was stable around the screw and increased the new bone volume in the histological analysis, but the smooth surface of the polymer and the prevention of corrosion product formation by the early corrosion resistance resulted in a low screw removal torque value. In other words, the PCL coating can provide a stent for the treatment of benign oesophageal stricture^40^.

As in the histological analysis results, stable and compact new bone formed on the PCL coating. The polymer layer was able to reduce the release of magnesium ions, thereby controlling the degradation rate of magnesium alloy in the *in vivo* environment^41^. Magnesium ions are strongly involved in bone metabolism and act as a protective factor against osteocyte proliferation-promoting factors and excessive bone resorption in the presence of magnesium^42^, but highly magnesium-substituted apatite has a toxic effect on bone cells and prevents the formation of the extracellular matrix^43^. Also, higher Mg ion concentrations inhibit the formation of hydroxyapatite crystals by binding to pyrophosphate to form insoluble salts^44^. The improved corrosion resistance of the PCL coating results in the release of a lower level of magnesium ions than that in the PEO group, which can lead to relatively more bone formation. Thus, although at the physiological level, magnesium can promote bone mineralization and regulate the growth of hydroxyapatite crystals^43^, the toxicity of the release of large amounts of magnesium ions into tissues must be considered. The presence of PCL on a bio-metallic material provides the potential for improving early angiogenesis of critical-sized bone defects by evaluating the proangiogenic factors using a human matrix and endothelial cells^45,46^. After 2 months of implantation, denser and thicker bone formed around the PCL-coated screw than the PEO-coated screw, which tended to be consistent with the results of other studies^41^. This feature is a great advantage in the application of polymer-coated implants for orthopaedic and maxillofacial surgeries because the slow release rate of magnesium ions and the high strength of polymer-coated implants allow enough time for bone healing and the promotion of new bone growth.

## Materials and Methods

### Material preparation

Pure Magnesium metal (rod-type as drawn, 99.9%, Goodfellow Cambridge Limited, England) was processed into a commercial screw, as shown Fig. 1(a). Although pure magnesium had low mechanical properties and corrosion resistance compared to alloys, which was only used to obtain the corrosion mechanism results between the polymer coating and the material. And it was chosen to suppress various variables such as the negative influence of the impurities in the alloy and the reaction between the additive element and the tissue.

Base and the reaction between the additive element and the tissue. Cortical screws were manufactured individually and machined to a length of 10 mm with a thread diameter of 2.4 mm.

### Surface treatment

In the first step of the PEO coating process, a platinum plate and a screw were placed to the cathode and anode, respectively, of a pulsed DC power supply (Kwangduk FA, Korea). The electrolyte used contained 1.0 M sodium hydroxide (NaOH), 0.10 M sodium phosphate (Na_3_PO_4_) and 0.1 M glycerol, and the process was conducted in constant current mode at 300 mA/cm^2^. The pulse width was set to 100 ms, and the duty cycle was fixed at 50% for 3 min.

In the second step of the PCL coating process, solutions with several concentrations of polycaprolactone (PCL) (molecular weight: 70,000–90,000, Sigma-Aldrich, USA) were prepared by dissolving the required amount of 5∼7 wt. % PCL in dichloromethane (Showa Chemical, Japan) under stirring for 2 h. The dip-coating method was performed by a dip coater (Micro Dip Coater EF-5100). The PEO-treated Mg screws were immersed and pulled out at a rate of 20 mm/min followed by drying at 25 °C for 10 min. The dip-coating procedure was repeated for 2∼6 cycles. The detailed conditions are shown in Fig. 1(c). The samples were dried at 40 °C for 24 h in a drying oven to remove the residual organic solvent and moisture.

### Characterization of surface morphology

The surface morphologies and cross-sections of the films obtained from the various treatments were observed by scanning electron microscopy (SEM) (JSM-6400, JEOL, Japan). The element concentrations in the film were determined through elemental mapping and line scanning by energy dispersive X-ray spectroscopy (EDX) (7274, Oxford Instruments, England). The film thickness of 5 screws was measured 3 times at the screw root, thread and flank points. The data were analysed for statistical significance using the one-way ANOVA test. *p* values less than 0.05 were considered significant.

### Corrosion behaviour in simulated body fluid

The 9 screws per group were implanted in an artificial bone model plate (Fig. 1(c)) to evaluate the wear behaviour when immersed in simulated body fluid (SBF) for 4, 8 and 12 weeks. The SBF was Hank’s balanced salt solution (H2387, Sigma Chemical Co, USA), which consisted of 0.185 g/l calcium chloride dihydrate, 0.09767 g/l magnesium sulfate, and 0.350 g/l sodium hydrogen carbonate at pH 7.4. In addition, the SBF was changed every 72 h to keep the concentrations of the ions constant, as is the case in body fluids. The artificial bone model plate was custom-made by Sawbones (Vashon, WA, USA) using a combination of materials consisting of a 10-mm-thick open-cell rigid foam and two short fibre-filled epoxy sheets (2 mm thick). The block and sheets were laminated together with 40.04 Kg/m^3^ solid rigid polyurethane foam. The mechanical properties of open-cell rigid foam are 15.0 Kg/m^3^ density, 0.67 MPa compressive strength, 53.0 MPa compressive modulus, and the short fibre-filled epoxy sheet have mechanical properties as 102 Kg/m^3^ density, 157 MPa compressive strength, 16.7 MPa compressive modulus (provided by the manufacturer). A 2.2-mm-diameter drill was used to make a hole with a depth of 8 mm.

The surface morphologies and cross-sections of the films prepared by the various treatments were observed by SEM. The element concentrations of the film were determined from element mapping and line scanning by energy dispersive EDX.

To evaluate the change in the volume of the 3 samples after the immersion test, the artificial bone model plate with the screw was scanned by a micro-CT system (SKYSCA 1076; Skyscan, Belgium) installed in the Center for University-Wide Research Facilities (CURF) at Chonbuk National University, South Korea. The reconstruction into 3D images was performed by the Bruker-Skyscan free software (®CTan, ®DataViewer, ®CTvox and ® CTvol). The new data set images of each case were reoriented using Data Viewer software, which gave the ability to reconstruct the screw and obtain virtual slices and volume-rendered reconstructions. Subsequently, the total volume of each screw was calculated by running the 3D analysis plugin of the CTAn software. Finally, 3D images were obtained using CTVox or CTvol software.

### *In vivo* test

A total of 8 male Sprague Dawley rats (270–280 g, Orient Bio Co., Korea) were used in this experiment, which was approved by the institution at which the experiments were performed and by the local animal ethics committee of Chonbuk National University, South Korea (IRB: CBNU 2017-0025). The experiment was performed in accordance with the relevant guidelines and regulations. During the experiments, 0.5 ml/100 g of ketamine (Ketamine HCl57.68 mg, Yuhan, Korea) was administered intramuscularly to the rats as anaesthesia. The PEO and PCL groups consisted of 16 screws per group. A 2.2-mm-diameter drill was used to make a hole with a depth of 8 mm in the tibia of both legs for histological tests and the femur for examining the removal torque. The screw was tightened to a torque of 10 Ncm using a digital torque gauge (9810 P, Aikoh Engineering Co., Japan) with 0.1 Ncm accuracy^47^. The rats in the PEO and PCL coating groups were sacrificed 4 and 8 weeks after implant placement, as shown in Fig. 1(d).

Analysis of the removal torque was performed with the screw implanted in the rat femur. The screw head in the femur was exposed, and the torque was measured using a digital torque gauge (9810 P, Aikoh Engineering Co., Japan). A reverse force was applied until complete rupture of the bone screw interface, and the force needed to cause displacement in the bone tissue was recorded. The data were analysed for statistical significance using the one-way ANOVA test. The *p* values less than 0.05 were considered significant. The surface morphology after removal of the screw was observed by SEM. The element concentrations in the film were determined through elemental mapping and line scanning by EDX.

The tibia sections containing the screw were cut and fixed in a 10% formalin solution for histological analysis. The harvested tibia was stained with the Villanueva osteochrome bone staining solution (Polysciences Inc., USA) and embedded in poly(methyl methacrylate). The embedded blocks were sectioned and ground along the longitudinal axis of the screw to a thickness of approximately 10–40 μm. Finally, histological analyses were performed to look for new bone between the tibia and the screw using optical microscopy (DM 2500 M, Leica, Germany). At 4 weeks and 8 weeks, new bone formation was observed by micro-CT (SKYSCAN 1076, Skyscan, Belgium) by setting a specific range around the screw, as shown in Fig. 1(e).
